# Supplementary material for: Genetic screens reveal a central role for heme metabolism in artemisinin susceptibility
Source: Nat Commun. 2020 Sep 23;11:4813. doi: 10.1038/s41467-020-18624-0 (PMC7511413; doi:10.1038/s41467-020-18624-0)
Supplement: Supplementary file 1 — Supplementary Information [file 41467_2020_18624_MOESM1_ESM.pdf]

SUPPLEMENTARY INFORMATION

Genetic screens reveal a central role for heme metabolism in artemisinin susceptibility

Clare R. Harding[1,2,8], Saima M. Sidik[1,8], Boryana Petrova[1,8], Nina F. Gnädig[3], John Okombo[3], Alice L. Herneisen[1], Kurt E. Ward[3,4], Benedikt M. Markus[1,5], Elizabeth A. Boydston[1], David A. Fidock[3,6], Sebastian Lourido[1,7]*

[1] Whitehead Institute for Biomedical Research, Cambridge, MA, USA

[2] Wellcome Centre for Molecular Parasitology, Institute of Infection, Immunity & Inflammation, University of Glasgow, Glasgow, UK

[3] Department of Microbiology and Immunology, Columbia University Irving Medical Center, New York, NY, USA

[4] Department of Microbiology and Immunology, University of Otago, Dunedin, New Zealand

[5] University of Freiburg, Faculty of Biology, Freiburg, Germany

[6] Division of Infectious Diseases, Department of Medicine, Columbia University Irving Medical Center, New York, NY, USA

[7] Biology Department, Massachusetts Institute of Technology, Cambridge, MA, USA

[8] These authors contributed equally to the work

* Correspondence: lourido@wi.mit.edu

SUPPLEMENTARY FIGURES & LEGENDS

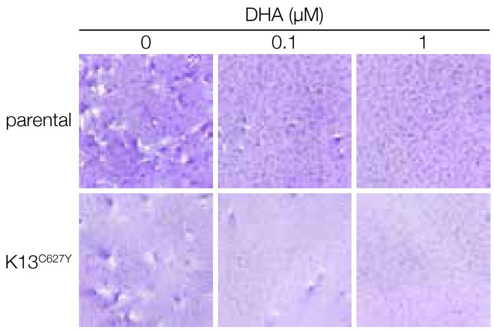

Supplementary Figure 1 I $K13^{C627Y}$ parasites proliferate normally and do not show DHA resistance in plaque assays. Plaque assay or parental and $K13^{C627Y}$ parasites, fixed after 7 days of drug treatment with the indicated concentration.

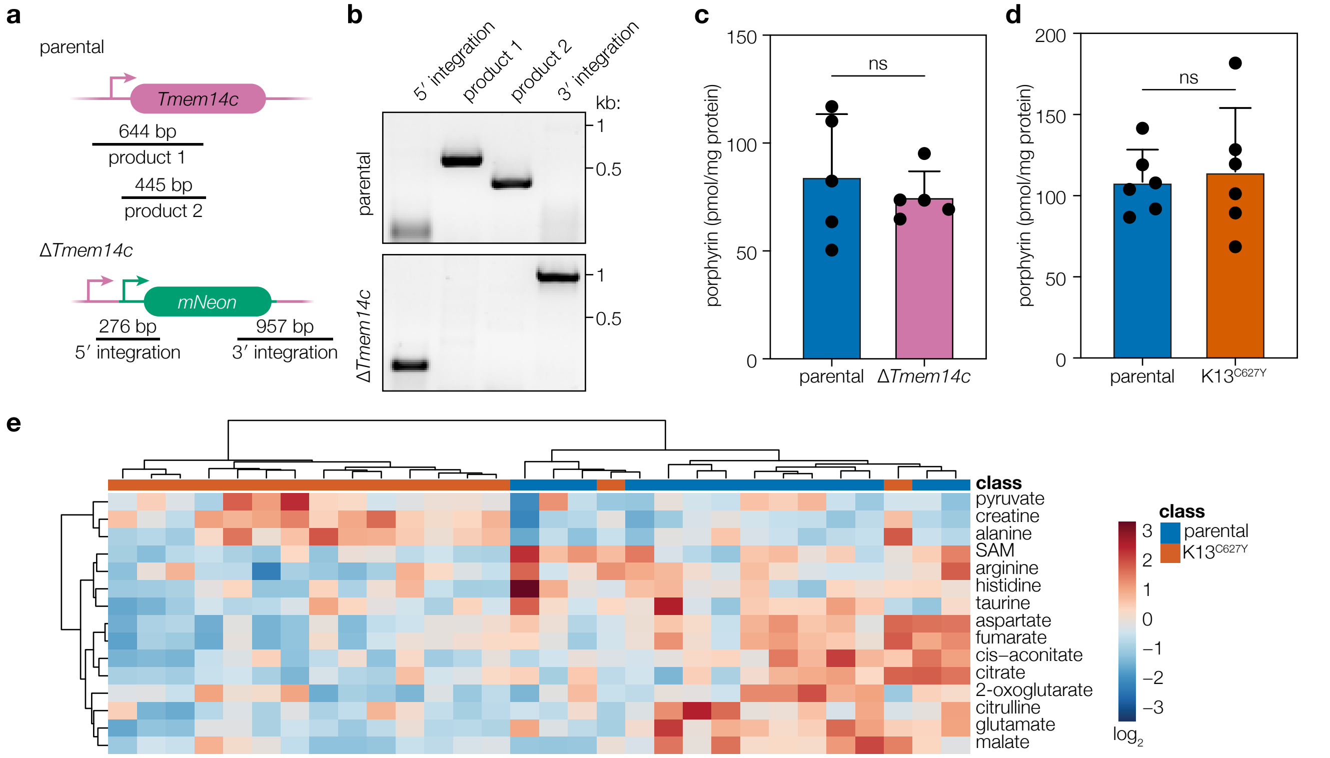

Supplementary Figure 2 I Construction of Δ*Tmem14c* and porphyrin measurements from Δ*Tmem14c* and $K13^{C627Y}$. a, Schematic showing strategy for creating Δ*Tmem14c* parasites. The coding sequence was replaced by an mNeonGreen cassette. **b**, PCR demonstrating correct deletion of *Tmem14c*. **c**, Total porphyrins were quantified from parental and Δ*Tmem14c* parasites. Results are mean ± SD for $n = 3$ independent experiments, each performed in technical duplicate. **d**, Total porphyrins were quantified from parental and $K13^{C627Y}$ parasites. Results are mean ± SD for $n = 3$ independent experiments; ns, not significant by two-tailed, unpaired t test. **e**, MetaboAnalyst heatmap analysis of top 15 changed polar metabolites between parental and $K13^{C627Y}$ parasites from 4 independent experiments. Metabolite peak areas were normalized to total metabolite signal (see **Methods**).

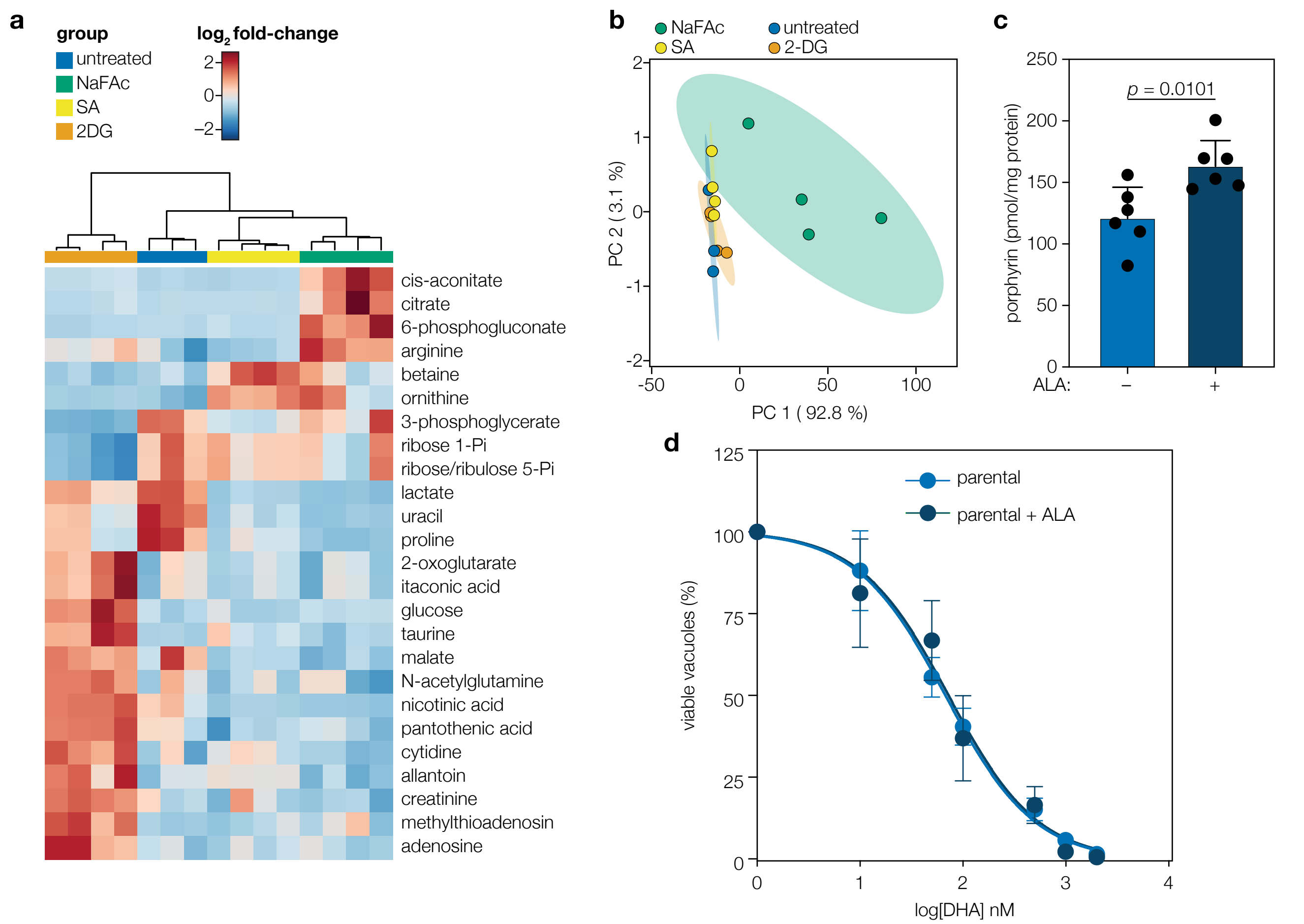

Supplementary Figure 3 | Effects of modulators of glycolysis, TCA, and heme biosynthesis on parasite metabolism. a, MetaboAnalyst heatmap analysis of the top 25 polar metabolites with altered abundance after the indicated treatment. Results are from a representative experiment performed in triplicate. Metabolite peak areas were normalized to total metabolite signal (see **Methods**). **b**, PCA plot indicating variance of total polar metabolites between various compound-treated samples. 500 mM NaFAc treatment had significant global effects on metabolite abundance, while 5 mM 2-DG and 10 mM SA had more subtle effects and overlapped with the parental, untreated line. **c**, Normalized total porphyrin levels in untreated or 200 μM ALA treated parasites. Results are mean ± SD of *n* = 3 independent experiments performed in duplicate; *p* values from two-tailed, unpaired t test on raw values. **d**, Dose-response curve for parasites treated with ALA or vehicle. No difference in the response to DHA could be seen (extra-sum-of-squares F test, *p* = 0.79). Results are mean ± SEM for *n* = 3 biological replicates.

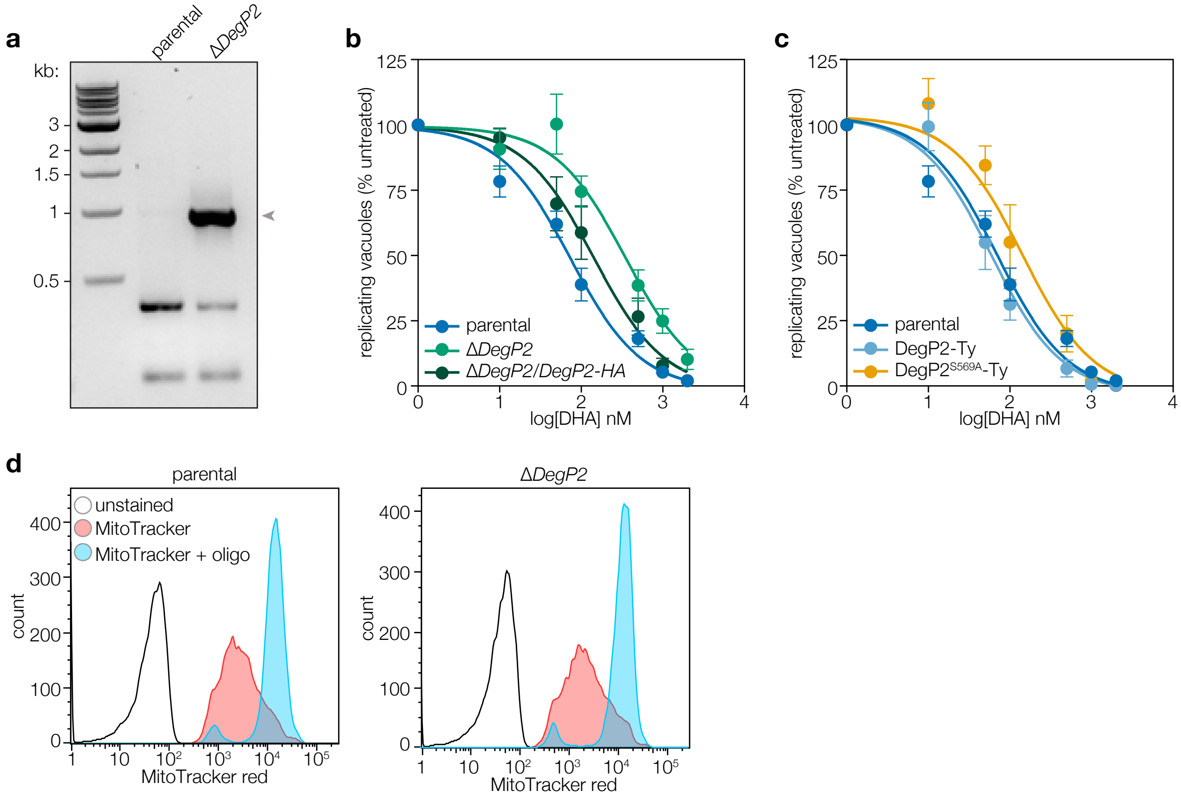

Supplementary Figure 4 | DegP2-deficient parasites are less susceptible to DHA but have normal mitochondrial polarization. a, PCR confirming loss of the endogenous locus and replacement with YFP. Arrow indicates the size of the PCR product expected after replacement. **b**, DHA dose-response curves. Results are mean ± SEM for *n* = 7, 7, or 4 independent experiments for the parental, Δ*DegP2*, or Δ*DegP2*/*DegP2-HA* strains, respectively. **c**, DHA dose-response curves. Results are mean ± SEM for *n* = 7, 4, or 4 independent replicates for the parental, DegP2-Ty, or DegP2^{S569A}-Ty strains, respectively. **d**, Histograms of flow cytometry data of MitoTracker-stained parental and Δ*DegP2* parasites, treated with oligomycin or untreated. No differences were observed between parental and Δ*DegP2* parasites. Results are representative of two independent experiments.

SUPPLEMENTARY TABLES

Supplementary Table 1. Summary of EC_{50} values.

Strain	Compound	Pre-treatment	Compound treatment duration (h)	Compound treatment time (h following invasion)	Intra/ extracellular	EC_{50} (nM)	95% CI	Figure
K13^{C627Y}	DHA		5		Extracellular	498	321.2–628.3	1e
parental	DHA		5		Extracellular	70	49.58–99.08	1e, 2d, 5d
K13^{C627Y}	DHA		5	1–6	Intracellular	1492	797.8–2059	1f
parental	DHA		5	1–6	Intracellular	548.5	372.3–772.6	1f, 2e
K13^{C627Y}	DHA		5	24–29	Intracellular	1123	885.3–1802	1f
parental	DHA		5	24–29	Intracellular	539.6	450.1–708.7	1g, 2e
Δ*Tmem14c*	DHA		5		Extracellular	36.9	27.44–49.37	2d
Δ*Tmem14c*	DHA		5	1–6	Intracellular	168	99–330.5	2e
Δ*Tmem14c*	DHA		5	24–29	Intracellular	77.4	66.79–89.32	2e
parental	DHA	2 h 500 µM NaFAc	5		Extracellular	271.3	174.7–417.5	3c
parental	DHA	2 h 10 mM SA	5		Extracellular	529.9	413.2–839.7	3d
parental	DHA	2 h 5 mM 2-DG	5		Extracellular	86.46	52.52–141.5	3e
parental	DHA	2 h vehicle	5		Extracellular	76.15	52.54–110.8	3c, 3d, 3e
parental	DHA	2 h 200 µM ALA	5		Extracellular	81.61	50.28–131.9	S3d
Δ*DegP2*	DHA		5		Extracellular	357.8	259.7–487.8	5d
Δ*DegP2*/ *DegP2-HA*	DHA		5		Extracellular	104.7	95.46–207.3	5d
DegP2-Ty	DHA		5		Extracellular	63	43.07–91.95	5d
DegP2^{S569A}-Ty	DHA		5		Extracellular	153.1	105.7–222.4	5d
Δ*DegP2*	TTFA		5		Extracellular	411	356–475	6c
Δ*DegP2*/ *DegP2-HA*	TTFA		5		Extracellular	264	223–316	6c
parental	TTFA		5		Extracellular	255	223–292	6c
ΔDegP2	ATQ		5		Extracellular	80	49.62–130.6	6d
parental	ATQ		5		Extracellular	69	43.32–112.1	6d

Supplementary Table 2. Summary of top hits from three replicates of the genome-wide screen. ns, not significantly enriched; [a](Sidik, 2016).

Gene ID	Annotation	Drug score			Phenotype score[a]
		Rep1	Rep2	Rep3	
TGGT1_290840	serine protease (DegP2)	9.97	3.9	7.03	-1.87
TGGT1_244200	α-ketoglutarate dehydrogenase (E1)	13.15	10.97	5.03	-4.8
TGGT1_219550	α-ketoglutarate dehydrogenase (E2)	12.01	12.26	4.99	-4.25
TGGT1_206470	pyruvate dehydrogenase (PDH-E3II)	12.9	12.55	9.07	-3.23
TGGT1_251680	Translationally controlled tumor protein (TCTP)	ns	2.65	3.24	-1.37
TGGT1_297080	pyridoxal kinase	5.63	ns	5.01	-0.41
TGGT1_272490	protoporphyrinogen oxidase	10.26	ns	9.66	-3.87
TGGT1_271410	hypothetical protein	2.59	ns	0.24	-1.1

Supplementary Table 3. MetaboAnalyst pathway analysis of polar metabolites from parental vs K13^{C627Y} parasites, normalized to mean parental values. Significantly different (FDR < 0.1) pathways are listed. Pathway impact values closer to 1 indicate higher node importance.

Pathway	Total compounds	Hits	*p* value	Holm's adjusted *p* value	FDR	Impact
Alanine, aspartate and glutamate metabolism	12	6	0.00026789	0.0075009	0.0075009	0.85185
Citrate cycle (TCA cycle)	20	7	0.0074721	0.20922	0.070083	0.30607
Glutathione metabolism	21	2	0.0096666	0.261	0.070083	0.20313
Porphyrin and chlorophyll metabolism	17	2	0.0096666	0.261	0.070083	0
