# Supplementary material for: Genetic screens reveal a central role for heme metabolism in artemisinin susceptibility
Source: Nat Commun. 2020 Sep 23;11:4813. doi: 10.1038/s41467-020-18624-0 (PMC7511413; doi:10.1038/s41467-020-18624-0)
Supplement: Supplementary file 3 — Reporting Summary [file 41467_2020_18624_MOESM3_ESM.pdf]

nature research

Corresponding author(s): Sebastian Lourido

Last updated by author(s): Aug 28, 2020

Reporting Summary

Nature Research wishes to improve the reproducibility of the work that we publish. This form provides structure for consistency and transparency in reporting. For further information on Nature Research policies, see our Editorial Policies and the Editorial Policy Checklist.

Statistics

For all statistical analyses, confirm that the following items are present in the figure legend, table legend, main text, or Methods section.

n/a	Confirmed	
☐	☒	The exact sample size (n) for each experimental group/condition, given as a discrete number and unit of measurement
☐	☒	A statement on whether measurements were taken from distinct samples or whether the same sample was measured repeatedly
☐	☒	The statistical test(s) used AND whether they are one- or two-sided<br>*Only common tests should be described solely by name; describe more complex techniques in the Methods section.*
☐	☒	A description of all covariates tested
☐	☒	A description of any assumptions or corrections, such as tests of normality and adjustment for multiple comparisons
☐	☒	A full description of the statistical parameters including central tendency (e.g. means) or other basic estimates (e.g. regression coefficient) AND variation (e.g. standard deviation) or associated estimates of uncertainty (e.g. confidence intervals)
☐	☒	For null hypothesis testing, the test statistic (e.g. F, t, r) with confidence intervals, effect sizes, degrees of freedom and P value noted<br>*Give P values as exact values whenever suitable.*
☒	☐	For Bayesian analysis, information on the choice of priors and Markov chain Monte Carlo settings
☒	☐	For hierarchical and complex designs, identification of the appropriate level for tests and full reporting of outcomes
☒	☐	Estimates of effect sizes (e.g. Cohen's d, Pearson's r), indicating how they were calculated

Our web collection on statistics for biologists contains articles on many of the points above.

Software and code

Policy information about availability of computer code

Data collection	Metabolomic data: XCalibur QuanBrowser 2.2 (ThermoFisher Scientific) and TraceFinder software (ThermoFisher Scientific)<br>Proteomic data: Proteome Discoverer 2.2 (ThermoFisher Scientific)<br>FACS data: BD CellQuest Pro v6.0<br>High content imaging: BioTech Gen8<br>Microscopy: NIS elements (Nikon)
Data analysis	Microscopy: Image J version 1.52p.<br>Mass Spectrometry: XCalibur QuanBrowser version 2.2 (Thermo Fisher Scientific) and TraceFinder software (ThermoFisher Scientific)<br>Data Analysis: Prism version 8.1.2 (GraphPad Software Inc.), MAGeCK (Li, et al. Genome Biology. 2014), MetaboAnalyst (ThermoFisher Scientific), Excel for Mac version 16.16.13 (Microsoft), CHOPCHOP (3; Lebun et al. 2019. Nucleic Acids Res)

For manuscripts utilizing custom algorithms or software that are central to the research but not yet described in published literature, software must be made available to editors and reviewers. We strongly encourage code deposition in a community repository (e.g. GitHub). See the Nature Research guidelines for submitting code & software for further information.

Data

Policy information about availability of data

All manuscripts must include a data availability statement. This statement should provide the following information, where applicable:

- Accession codes, unique identifiers, or web links for publicly available datasets
- A list of figures that have associated raw data
- A description of any restrictions on data availability

CRISPR screening raw data are available through the Gene Expression Omnibus with the accession number GSE153785[https://www.ncbi.nlm.nih.gov/geo/query/

acc.cgi?acc=GSE153785]. Minimally processed CRISPR screen data are available in Supplementary Data 1. Raw thermal proteome profiling data is available through the ProteomeXchange Consortium via the PRIDE partner repository with the dataset identifier PXD019917[https://www.ebi.ac.uk/pride/archive/projects/PXD019917]. Minimally processed thermal proteome profiling data is available in Supplementary Data 5. Raw metabolomics data are available in Supplementary Data 2, 4, and 6. Additional raw data is included in the Source Data File. Additional data is available from the authors upon request. Toxoplasma genome information can be found in ToxoDB[https://toxodb.org].

Field-specific reporting

Please select the one below that is the best fit for your research. If you are not sure, read the appropriate sections before making your selection.

☒ Life sciences ☐ Behavioural & social sciences ☐ Ecological, evolutionary & environmental sciences

For a reference copy of the document with all sections, see nature.com/documents/nr-reporting-summary-flat.pdf

Life sciences study design

All studies must disclose on these points even when the disclosure is negative.

Sample size	Sample sizes were not predetermined. Experiments were generally repeated at least three times. When additional experiments were performed all experiments are reported. Sample sizes are consistent with similar published studies.
Data exclusions	No exclusion criteria were predetermined and no data were excluded.
Replication	All results in the manuscript were replicated at least three times, independently. Where there was variability between replicates (e.g. in the CRISPR screens) this has been recorded. All experiments performed have been reported where they contribute to a statistical calculation.
Randomization	Assignment of strains to treatment groups was randomized between biological replicates.
Blinding	Investigators were not blinded during data acquisition and analysis. The analyses performed have quantitative endpoints and are not subject to investigator bias.

Reporting for specific materials, systems and methods

We require information from authors about some types of materials, experimental systems and methods used in many studies. Here, indicate whether each material, system or method listed is relevant to your study. If you are not sure if a list item applies to your research, read the appropriate section before selecting a response.

Materials & experimental systems

n/a	Involved in the study	
☐	☒	Antibodies
☐	☒	Eukaryotic cell lines
☒	☐	Palaeontology and archaeology
☒	☐	Animals and other organisms
☒	☐	Human research participants
☒	☐	Clinical data
☒	☐	Dual use research of concern

Methods

n/a	Involved in the study	
☒	☐	ChIP-seq
☒	☐	Flow cytometry
☒	☐	MRI-based neuroimaging

Antibodies

Antibodies used	mouse anti-HA (Biolegends 901513), 1:1000 (IFA), 1:5000 (IB); mouse anti-Ty, 1:1000 (IFA); rabbit anti-mtHSP70, 1:1000 (IFA), guinea pig anti-CDPK1, 1:5000 (IFA), 1:20000 (IB); Alexa-Fluor-labeled secondary antibodies (ThermoFisher), 1:1000 (IFA); IR secondary antibodies (LiCor), 1:10000 (IB)
Validation	mouse anti-HA (Biolegends 901513): Hogarth CA, Evans E, Onken J, et al. CYP26 Enzymes Are Necessary Within the Postnatal Seminiferous Epithelium for Normal Murine Spermatogenesis. Biol Reprod. 2015;93(1):19. doi:10.1095/biolreprod.115.129718<br><br>mouse anti-Ty: Bastin, P., Bagherzadeh, Z., Matthews, K. R. & Gull, K. A novel epitope tag system to study protein targeting and organelle biogenesis in Trypanosoma brucei. Mol. Biochem. Parasitol. 77, 235–239 (1996).<br><br>rabbit anti-mtHSP70: Pino, P. et al. Dual targeting of antioxidant and metabolic enzymes to the mitochondrion and the apicoplast of Toxoplasma gondii. PLoS Pathog. 3, e115 (2007).<br><br>guinea pig anti-CDPK1: Waldman, B. S. et al. Identification of a Master Regulator of Differentiation in Toxoplasma. Cell 180, 359–372.e16 (2020).

Bands were seen at the correct molecular weights for all antibodies. For mouse anti-Ty, bands were only seen in lines known to contain the epitope tag. For anti-CDPK1, bands were absent in a CDPK1 KO parasite line.

Eukaryotic cell lines

Policy information about cell lines

Cell line source(s)	HFF-1 (ATCC® SCRC-1041™). Toxoplasma gondii lines used or created in this study as described in the Methods based on the following published strains:<br>RH/Cas9: Markus, B. M., Bell, G. W., Lorenzi, H. A. & Lourido, S. Optimizing Systems for Cas9 Expression in Toxoplasma gondii. mSphere vol. 4 (2019).<br>RH Δku80: Huynh, M.-H. & Carruthers, V. B. Tagging of endogenous genes in a Toxoplasma gondii strain lacking Ku80. Eukaryot. Cell 8, 530–539 (2009).<br>DiCre: Hunt, A. et al. Differential requirements of cyclase associated protein (CAP) for actin turnover during the lytic cycle of Toxoplasma gondii. bioRxiv 569368 (2019) doi:10.1101/569368.
Authentication	HFFs were authenticated by the ATCC using intraspecies STR analysis. Toxoplasma strains were genotyped by PCR and sequencing, as appropriate.
Mycoplasma contamination	HFF human cells and parasites were frequently tested and found negative for Mycoplasma.
Commonly misidentified lines (See ICLAC register)	No commonly misidentified lines were used in this study.
