# Supplementary material for: Genetic screens reveal a central role for heme metabolism in artemisinin susceptibility
Source: Nat Commun. 2020 Sep 23;11:4813. doi: 10.1038/s41467-020-18624-0 (PMC7511413; doi:10.1038/s41467-020-18624-0)
Supplement: Supplementary file 4 — Description of Additional Supplementary Files [file 41467_2020_18624_MOESM4_ESM.pdf]

Description of Additional Supplementary Files

File Name: Supplementary Data 1
Description: Full CRISPR screen data.

File Name: Supplementary Data 2
Description: Raw and normalized data from metabolomics from ΔTmem14c and its parental line

File Name: Supplementary Data 3
Description: **Hits from MAGeCK analysis**. All genes significantly (defined as FDR

File Name: Supplementary Data 4
Description: Raw data from untargeted polar metabolomics from parental lines treated with metabolic inhibitors (NaFAc, SA, 2-DG).

File Name: Supplementary Data 5
Description: Protein abundance in TPP samples relative to abundance in the 37° C sample.

File Name: Supplementary Data 6
Description: Raw data from untargeted polar metabolomics from parental and ΔDegP2 parasite lines. Δ

File Name: Supplementary Data 7
Description: *T. gondii* and *P. falciparum* primers used in this study.
